# Integrative analysis of purine metabolites and gut microbiota in patients with neuromyelitis optica spectrum disorders after mycophenolate mofetil treatment

**DOI:** 10.1186/s12883-023-03500-3

**Published:** 2023-12-16

**Authors:** Gong Li, Xiaoyu Ma, Lijuan Xia, Ran Wei, Xiran Wang, Cang Li, Yuge Wang, Limin He, Hao Ren, Jian Sun, Wei Qiu

**Affiliations:** 1 College of veterinary medicine, South China Agricultural University, Guangzhou, China; 2 Department of Neurology, The Second Hospital of Shandong University, Jinan, 250033 China; 3 Department of Neurology, The Third Affiliated Hospital of Sun Yat-sen University, No. 600 Tianhe Road, Guangzhou, 510630 China

**Keywords:** Neuromyelitis Optica Spectrum Disorders, Mycophenolate Mofetil, Gut Microbiota, Therapeutic Efficacy, PSP Metabolites

## Abstract

**Background:**

Neuromyelitis optica spectrum disorder (NMOSD) is a recurring inflammatory demyelinating disease that is commonly observed in Asian countries like China. Prior investigations have shown that mycophenolate mofetil (MMF) with better biocompatibility compared to azathioprine (AZA), and can prevent relapses of NMOSD, but the efficacy was controversially reported in different NMOSD cases. We aimed to explore the factors that weaken efficacy of MMF in NMOSD.

**Methods:**

A total of 34 NMOSD patients treated with MMF were prospectively enrolled and grouped according to the therapeutic efficacy as effective group (EG, n = 23) versus less-effective group (LEG, n = 11). The purine metabolites were profiled in serum samples and gut microbiota was analyzed using 16S rRNA sequencing with stool samples from the same patients.

**Results:**

Purine salvage pathway (PSP) metabolites (inosine, hypoxanthine, xanthine, guanine and uric acid) in the serum of NMOSD patients were elevated in the LEG compared to EG (*p* < 0.05). Additionally, the richness and microbial diversity of gut microbiota was found to be similar between EG and LEG patients. However, LEG patients had increased presence of *Clostridium* and *Synergistes* but decreased abundance of the *Coprococcus* genus.

**Conclusions:**

The PSP metabolites and composition of the gut microbiota were changed between patients with or without optimal clinical response after MMF treatment. This may help us to understand the pharmacodynamics of MMF in NMOSD.

## Introduction

Neuromyelitis optica spectrum disorders (NMOSD) is a severe, disabling autoimmune disease of the central nervous system (CNS) characterized by its association with serum anti-aquaporin-4 antibodies (AQP4-IgG) [1–4]. Clinically, NMOSD results in a range of syndromes of varied severities including recurrent paralysis, blindness and brain syndromes [5]. Immunosuppressive therapies including administration of azathioprine (AZA) and mycophenolate mofetil (MMF) in addition to the new B cell- and complement-targeted therapies are the current NMOSD treatments aimed at relapse prevention [6, 7], with MMF demonstrating better tolerability compared to AZA [8]. MMF is a prodrug whose active metabolite mycophenolic acid (MPA) inhibits inosine monophosphate dehydrogenase (IMPDH) and suppresses the de novo synthesis of guanine nucleotides in rapidly-proliferating cells such as T and B lymphocytes [9, 10]. MMF/MPA treatments significantly and comprehensively lower the levels of baseline purine metabolites in intestinal cell models as well as in patients [11, 12]. However, previous studies have shown mixed efficacy of MMF in treating NMOSD, the proportion of recurrence-free patients in NMOSD is 46–73%, the Expanded Disability Status Scale (EDSS) score decreased or stabilized in 83–91% of patients [6, 13–15].

Our laboratory recently demonstrated that the gut microbiomes of healthy individuals can be distinguished from NMOSD patients. In particular, the degree of *Streptococcus* spp. colonization was significantly and positively correlated with disease severity [16]. Other studies have demonstrated strong associations between several *Clostridium* spp. and NMOSD development [17, 18]. These studies linked dynamic interactions of the host gut microbiota to NMOSD. However, it is not known whether NMOSD therapies such as MMF administration are also influenced by the gut microbiota and whether the gut microbiota has a role in the efficacy of these therapies.

In the current study, we performed metabolic profiling and microbiome analysis on samples collected from NMOSD patients. This may not only help us to understand the pharmacodynamics of MMF but may also reveal the reasons of therapeutic effect.

## Materials and methods

### Reagents and chemicals

Inosine, hypoxanthine, xanthine, guanine and uric acid were obtained from MedChemExpress (Shanghai, China) and were prepared as 1000 ppm methanol stocks. NaOH 10 mM was used as necessary to increase solubility. Acetonitrile was purchased from Macklin (Shanghai, China).

### Patient enrollment

The study group included 34 NMOSD patients who had received MMF therapy (0.5 g MMF orally, twice a day for long term therapy) at the Third Affiliated Hospital of Sun Yat-sen University (Guangzhou, China). The primary inclusion criteria were (1) clinical diagnosis of NMOSD and seropositive for AQP4-IgG [19, 20]; (2) patients aged 15–65 and (3) an Kurtzke Expanded Disability Status Scale (EDSS) score ≤ 8.0. Patients who met any of the following key criteria were excluded: (1) treatment with antibiotics or probiotics within the last 3 months; (2) emergency treatment such as plasmapheresis or intravenous methylprednisolone for acute recurrence within the last 1 month; (3) Concurrent disease with diabetes mellitus, systemic autoimmune diseases or other neurological diseases.

Stool and serum samples were taken from the patient that received MMF treatment at least for 3 months. Stool samples were taken in the morning. Serum samples were taken for 0.5, 1 and 2 h after receiving MMF in the early morning. Clinical data was collected included relapses, follow-up time and EDSS score. The 34 participants were divided into two groups: patients with no relapse were classified into the effective group (EG). Patients who relapsed after 3 months of MMF treatment were treated as the less-effective group (LEG). This study was approved by the ethics Committee of the Third Affiliated Hospital of Sun Yat-sen University ([2020]02-016-01) and all patients signed informed consent.

### HPLC-MS/MS analysis

Serum sample processing and HPLC-UV–MS/MS analysis were performed as previously described with minor modifications [21]. In brief, chromatographic separations were conducted using a Shimadzu LC-30AD series HPLC system (Shimadzu, Kyoto, Japan) with an Frulic N column (AZYP, LLC, Arlington, USA). Analytes were eluted at a flow rate of 0.4 mL/min using a gradient of phase A (0.2% formic acid) and phase B (0.2% formic acid in acetonitrile) as follows: 0–0.5 min, 80% B; 0.5–4.5 min, 50% B; 4.5–8 min, 80% B. The eluates were monitored by UV at 254 nm and MS detection in parallel. The injection volume as 5 µL, and the column temperature was maintained at 23 °C. MS detection was performed using an AB Sciex Triple Quad 5500 (AB Sciex, Toronto, Canada).

### Microbiome 16S rRNA gene sequencing, alignment and analysis

Microbial DNA was extracted from fecal samples of NMOSD patients using the E.Z.N.A. Stool DNA Kit (Omega Bio-Tek, Norcross, GA, USA) according to the manufacturer’s protocol. Extracted DNA was qualitatively and quantitatively analyzed by gel electrophoresis and ultraviolet spectroscopy before sequencing. Thereafter, bacterial 16S rDNA V3–V4 regions were amplified using primers 341 F and 806R (Illumina, San Diego, CA, USA). The resulting amplicons were sequenced using the Illumina NovaSeq 6000 platform (Guangdong Longsee Biomedical, Foshan, China).

### Statistical analyses

The data were expressed as the mean ± standard deviation (SD). A unpaired parametric t-test was performed for comparison of the two groups using Prism v8.0 software (GraphPad, San Diego, CA, USA). A *p* value of 0.05 was considered to be statistically significant.

## Results

### Clinical characteristics

A total of 34 NMOSD patients who had received MMF therapy were recruited. All patients were diagnosed with NMOSD according to clinical diagnosis and seropositive for AQP4-IgG [19]. Immunotherapies such as MMF or AZA typically require 3–6 months to reach a milestone of stable effectiveness [22]. Hence, all the recurrences we recorded in this study occurred after 6 months of MMF use. The acute treatment guideline for NMOSD involves high-dose steroid therapy, follow with a gradual reduction in steroid usage and long-term maintenance [22–25]. Throughout the steroid treatment process, patients were gradually tapered to a maintenance dose of 8 mg methylprednisolone or 10 mg prednisone every other day or 5 mg prednisone once daily during the remission phase. As 4 mg methylprednisolone is equal to 5 mg prednisone, they all use similar doses of steroids. And in the remission stage, they all didn’t use other immunotherapy to prevent relapse currently and previously.

Our group of NMOSD patients were analyzed according to their clinical responses to MMF therapies and grouped according to responders (EG) and less-responders (LEG). The clinical characteristics of the patients are shown in Table 1. Significant differences in relapse times (*p* < 0.001) and EDSS after MMF (*p* = 0.005) were observed between EG and LEG patients.

**Table 1:** Clinical parameters in NMOSD patients according to response to MMF

Variables	EG (n = 23)	LEG (n = 11)	*p* value
Relapse times	0	2.00 ± 0.89	< 0.001
Age (years)	39.95 ± 12.20	47.45 ± 11.76	0.106
Gender (male: female)	5:18	1:10	0.638
Follow-up time (months)	21.00 ± 14.34	37 ± 20.27	0.060
EDSS before MMF	4.00 ± 0.86	3.5 ± 1.06	0.228
EDSS after MMF	3.00 ± 1.06	4.0 ± 1.30	0.005

### Purine salvage pathway metabolites are maintained at high levels in LEG patients

**Fig. 1:**
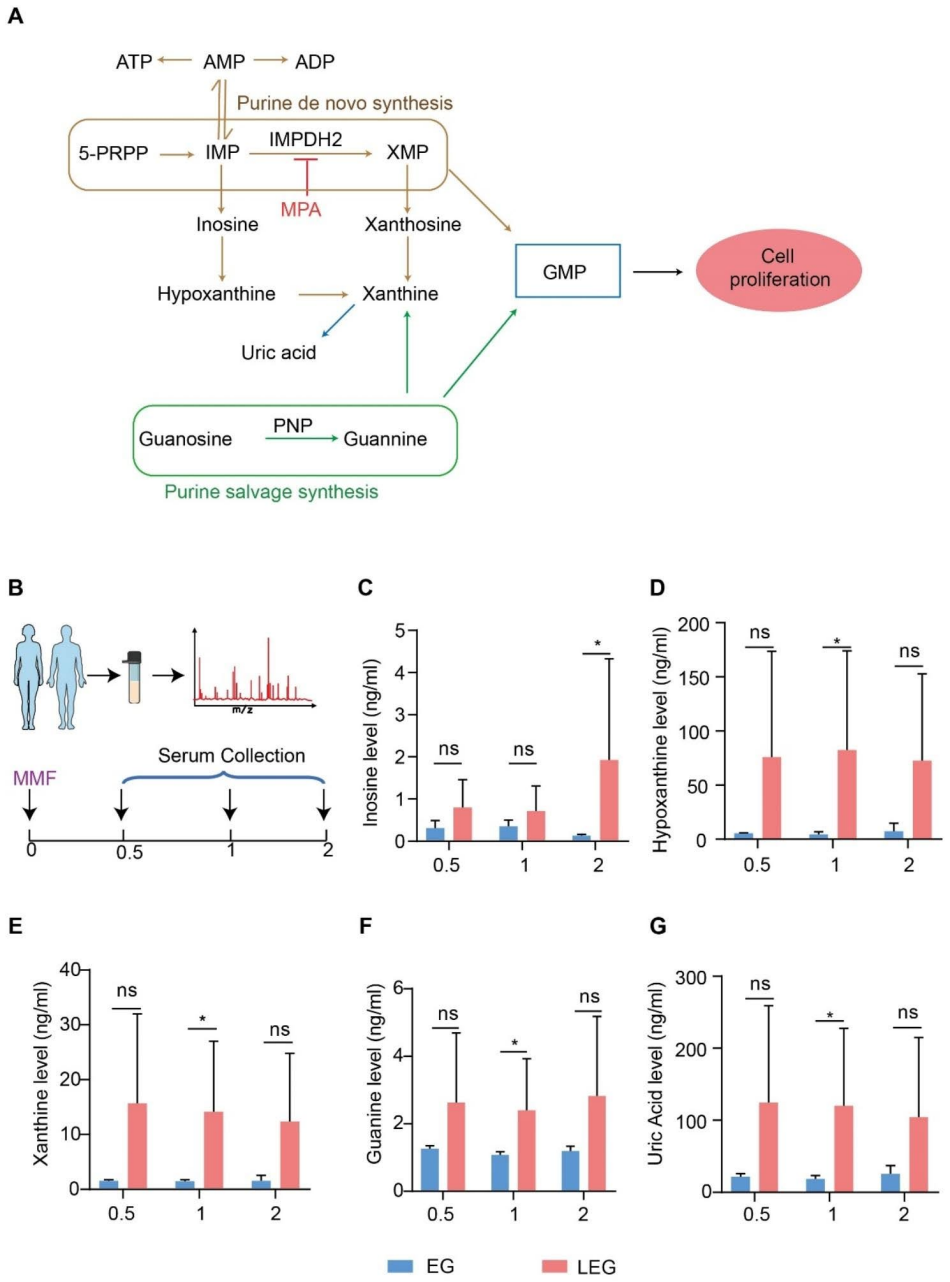
Purine salvage pathway metabolites are maintained at high levels in LEG patients. (**A**) Schematic of the de novo and salvage pathways of purine synthesis. (**B**) Serum collection point for the experiments. Quantification of the levels of (**C**) Inosine (**D**) Hypoxanthine (**E**) Xanthine (**F**) Guanine and (**G**) Uric acid. *p < 0.05

We examined purine metabolites whose biosynthesis is targeted by MMF(MPA) in the serum of recruited patients (Fig. 1A-B). Interestingly, we found distinct differences in the abundance of the purine salvage pathway (PSP) metabolites inosine, hypoxanthine, xanthine, guanine and uric acid (UA) for the patients that responded to MMF therapy (EG) and non-responders (LEG). These 5 PSP metabolites were all elevated in the serum of LEG patients. In particular, inosine, hypoxanthine, xanthine and guanine were significantly (*p* < 0.05) decreased in EG patients 1 h after receiving the MMF. At 2 h following MMF intervention, inosine levels reached the level of significance (*p* < 0.05) and were also lower in EG patients (Fig. 1C-G). The observations indicated a negative correlation between the MMF therapeutic response and PSP metabolite levels in serum of patients.

### Altered gut microbiota in NMOSD patients by their responses to MMF therapy

**Fig. 2:**
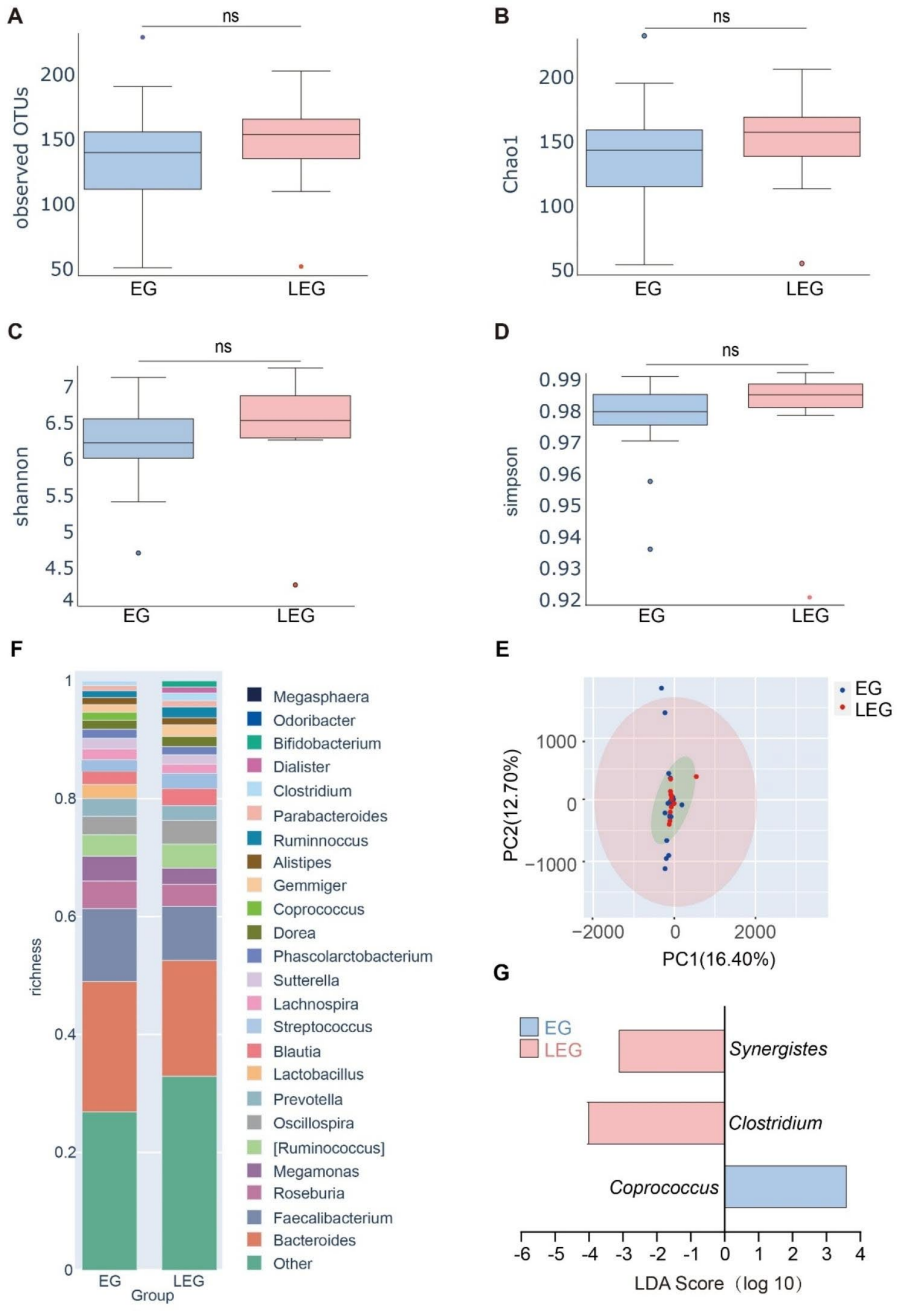
Altered gut microbiota in NMOSD patients by their responses to MMF therapy. (**A**) Observed OTUs. (**B**) Chao1 index. (**C**) Shannon index. (**D**) Simpson index of the gut microbiota in EG and LEG samples. (**E**) Principal component analysis. (**F**) Composition of the gut microbiota at the genus levels in fecal samples in EG and LEG samples. (**G**) LEfSe (Linear discriminant analysis effect size) analysis showing taxa associated with therapeutic response of MMF. n = 23 for effective group (EG) and n = 11 for less-effective group (LEG). *p < 0.05

Previous studies have indicated that the gut microbiota participates in the development and pathogenesis of NMOSD [16–18, 26]. The gut microbiota was profiled for each patient using 16 S rRNA sequencing. The microbial richness (operational taxonomic units, OTUs and Chao1 indices) and microbial diversity (Shannon and Simpson indices) were similar between EG and LEG patients (Fig. 2A-D). β diversity was assessed using principal-coordinate analysis (PCA) based on weighted UniFrac metrics and the results again indicated no compositional alterations between the microbiota of the patients in the two groups (Fig. 2E).

Since no structural shifts were observed among the patients in the response to MMF, we extended our taxonomic comparisons. The microbial compositions of each group were compared using the 20 most-abundant operational taxonomic units OUT at the genus level. Overall, *Bacteroides* dominated in both EG and LEG patients while the other dominate genera including *Faecalibacterium*, *Roseburia* and *Megamonas*, rarely exceeded 30% of all sequences (Fig. 2F). Interestingly, LEfSe analysis indicated that the abundance of *Clostridium* and *Synergistes* had significantly increased for EG and corresponded to a significant decline in abundance of the *Coprococcus* genus in LEG (Fig. 2G). These results indicated that overall, the gut microbiota stayed compositionally stable in the MMF therapy while the abundance of 3 bacterial genera were significantly altered.

## Discussion

NMOSD pathogenesis has been linked to the activity of microbiota [26, 27]. Long-term MMF therapy is widely used in immunological and NMOSD diseases although not all patients display the optimal clinical response. Whether the microbiota is also involved in the therapeutic response to the drugs against NMOSD has not been previously investigated. In this study, our findings first showed that the purine metabolites and composition of the gut microbiota were changed.

The NMOSD patients may experience pathogenesis of different level that influenced by various factors such as disease duration, comorbidities, prior disease-modifying treatment (DMT), and their baseline magnetic resonance imaging (MRI) status. However, NMOSD exacerbation is often reported to be associated with recurrences rather than a gradual progression, typically manifesting as a “cliff-like” exacerbation followed by incomplete recovery [6]. Consequently, the worsening of NMOSD appears to have little correlation with disease duration [7]. Based on our pre-survey, all patients recruited in our study had no histories for other DMT. Since the MMF has been primarily considered to target the relapses of NMOSD, we assessed failed responses of patients to MMF by examining recurrences and the EDSS.

The assessment of the effectiveness of MMF primarily relies on the area under the plasma concentration-time curve from 0 to 12 h (AUC_0 − 12_) as the principal parameter. However, while the AUC_0 − 12_ of the MPA concentration-time curve exhibits a strong correlation with pharmacological effects, its measurement proves to be arduous in a clinical setting. Several clinical trials have demonstrated that the predicted value of the AUC from 0 to 2 h (AUC_0 − 2_) can effectively evaluate the AUC from 0 to 12 h [28, 29]. Considering the challenges associated with sampling during clinical practice, we have chosen to employ three specific time points (0.5, 1, and 2 h) to represent the aforementioned parameter. We found that the levels of four PSP metabolites, including inosine, hypoxanthine, xanthine and guanine were significantly decreased in EG patients 1 h after receiving the MMF compared to LEG patients. MMF was originally designed to inhibit GMP and GTP synthesis. However, GTP synthesis is also coupled to purine *de novo* synthesis via PRPP availability that is regulated by the rapamycin complex 1 (mTORC1) [30]. Thus, other purines and in particular the PSP metabolites should be suppressed by the presence of MMF. In support of this, previous studies have demonstrated that levels of PSP metabolites levels in serum or plasma elevated in patients with CNS disease [31, 32]. Similarly, UA levels were mildly elevated in NMOSD patients during relapse compared with patients with a good prognosis [33].These findings coupled with our results potentially indicated that selected PSP metabolites might be used to distinguish the therapeutic efficacy of MMF in NMOSD patients.

A key factor for the poor response to MMF therapy in NMOSD patients has been linked to host genetic polymorphisms. For instance, single nucleotide polymorphisms (SNPs) in the genes encoding the MPA metabolizing enzyme UDP-glucuronosyltransferase 1–9 (UGT1A9) or the molecular targets (IMPDH1 and IMPDH2) are linked to a weakened drug responsiveness of individual patients [19, 30, 34]. In addition to SNP differences, the metabolic capacity of gut microbiota also actively participates in the utilization and metabolism of MMF e.g. through β-glucuronidase (GUS) enzymes expressed by gut commensals [35–37]. We profiled the microbiomes of NMOSD patients that were grouped according to their therapeutic responses to MMF therapy. Out of expectation, significant changes were not found in the microbial richness and diversity in the MMF non-responders compared to responders in this study. This implied a minor impact of the established host microbiome on the therapeutic response to MMF.

Currently, numerous studies have demonstrated that patients with NMOSD undergo substantial alterations in the composition of their gut microbiota following administration of immunotherapy drugs. Cree et al. observed a noteworthy proliferation of *Clostridium perfringens* in NMOSD patients treated with rituximab, in comparison to a control group [38]. In a separate study, Junli Gong et al. revealed a decrease in the relative abundance of the *Streptococcus* genus following treatment with immunosuppressants (MMF and AZA), when compared to an untreated group [39]. These findings suggest a potential association between the enrichment of *C. perfringens* and *Streptococcus* in NMOSD and the development of the disease. In this study, the abundance of *Clostridium* and *Synergistes* had significantly increased for EG patients and corresponded to a significant decline in abundance of the *Coprococcus* genus in LEG patients. *Clostridium* in the Firmicutes phylum was related to the AQP4-IgG–associated NMOSD [17, 18]. Moreover, the genus *Coprococcus* includes species that are strong butyrate producers [40–42] so that one possible explanation for our results is that they alleviate the syndromes of NMOSD by butyrate-dependent anti-inflammatory effects as well as having a negative effect on IL-17 A expression [43, 44]. A recent study also spotlighted this genus, whose abundance sharply reduced in NMOSD patients in comparison with the health control [45]. Hence, the existing *Coprococcus* species might be beneficial microbes to patients with NMOSD and ameliorate its symptoms by curtailing the levels of pro-inflammatory cytokines. This is an encouraging finding, and further investigation is needed to identify the mechanism adopted by *Coprococcus* couple with MMF to suppress the process of NMOSD development.

However, there are several limitations for this study. First, the absence of criteria for assessing the effectiveness of NMOSD treatment may weaken our conclusion. Our analysis based on relapse frequency and EDSS as criteria may introduce inherent bias into our findings. Second, the limited sampling size restricts our capacity to establish comprehensive conclusive remarks. Also, this precluded the consideration of baseline MRI status and general complications.

Future investigations would incorporate a comprehensive categorization of patients’ conditions, which is beneficial to better understand NMOSD.

## Conclusions

In this study, altered PSP metabolites and composition of the gut microbiota were observed between NMOSD patients with or without optimal clinical response after MMF treatment. Poor clinical response might be the increased abundance of *Clostridium* and *Synergistes* and decreased relative abundances of the genera *Coprococcus.* Our data might help to understand the pharmacodynamics of MMF in the aspect of the gut-brain axis. Furthermore, in view of the fact that MMF is so widely used in NMOSD, further studies investigating the mechanism of this *Coprococcus in* NMOSD are needed.

## Data Availability

The raw sequence data from the fecal microbiota in this paper were uploaded to the Sequence Read Archive (SRA) data (https://www.ncbi.nlm.nih.gov/sra) under accession number PRJNA813001.

## Abbreviations

MMF: Mycophenolate Mofetil
NMOSD: Neuromyelitis Optica Spectrum Disorders
EG: Effective Group
LEG: Less-Effective Group
PSP: Purine Salvage Pathway
MPA: Mycophenolic Acid
AQP4-IgG: Anti-Aquaporin-4 Antibodies
EDSS: Expanded Disability Status Scale
SNPs: Single Nucleotide Polymorphisms:UGT1A9:UDP-Glucuronosyltransferase 1–9
IMPDH: Inosine Monophosphate Dehydrogenase
SNP: Single Nucleotide Polymorphisms
GUS: β-glucuronidase
CNS: Central Nervous System
